# “An Important Part of Who I am”: The Predictors of Dietary Adherence among Weight-Loss, Vegetarian, Vegan, Paleo, and Gluten-Free Dietary Groups

**DOI:** 10.3390/nu12040970

**Published:** 2020-04-01

**Authors:** Tegan Cruwys, Rebecca Norwood, Veronique S. Chachay, Evangelos Ntontis, Jeanie Sheffield

**Affiliations:** 1 Research School of Psychology, The Australian National University, Canberra ACT 2601, Australia; 2 School of Psychology, The University of Queensland, Brisbane QLD 4072, Australia; rebecca.norwood@uq.net.au (R.N.); j.sheffield@psy.uq.edu.au (J.S.); 3 School of Human Movement and Nutrition Sciences, The University of Queensland, Brisbane QLD 4072, Australia; v.chachay@uq.edu.au; 4 School of Psychology, Politics, and Sociology, Canterbury Christ Church University, Canterbury CT1 1QU, Kent, UK; evangelos.ntontis@canterbury.ac.uk

**Keywords:** adherence, food choice, restrictive diets, dietary motivation, social identity, self-efficacy

## Abstract

Weight-loss diets are notorious for their low adherence, which is a barrier to efforts to reduce population rates of overweight and obesity. However, there is some evidence that adherence is better among people on other kinds of diets, such as vegan and gluten free. This study aimed to explore the predictors of dietary adherence across five restrictive dietary patterns (vegan, vegetarian, paleo, gluten free, and weight loss). This study used both qualitative and quantitative methods among 292 adult community members who were following a restrictive dietary pattern. Personality, mental health, and motivational predictors of adherence were examined. Substantial differences in adherence were found between dietary groups, with vegans and vegetarians being particularly high in adherence and gluten-free and weight-loss dieters being comparably low. Four consistent predictors of adherence across different dietary patterns were supported in both the quantitative and qualitative analyses. Self-efficacy and social identification with one’s dietary group positively predicted adherence. Conversely, being motivated in one’s dietary choices by mood or by weight control negatively predicted adherence. These findings speak to the importance of social and motivational factors in determining adherence. The results also illustrate the utility of looking beyond weight-loss dieters and virtuous individual traits for insights into how adherence may be improved.

## 1. Introduction

Overweight and obesity have been central to the global public health agenda in the last two decades [1]. The most likely intervention that will be undertaken by a person with overweight or obesity is dietary restriction, often on the recommendation of a health professional [2]. However, decades of research indicates that weight-loss diets typically fail [3,4]. Although a minority of people succeed in achieving short term weight loss, over the longer term, people who attempt to lose weight are more likely to gain weight over time than their nondieting counterparts [5]. This has fueled a growing body of research into the factors that support dietary adherence. However, almost all of this research has focused on people attempting weight loss. What has received comparatively little attention is the predictors of adherence among people on other kinds of restrictive dietary patterns: for example, vegan, vegetarian, paleo, and gluten free. People following these dietary patterns are, at least anecdotally, able to adhere for many years. This is despite the fact that these diets may be more restrictive than weight-loss diets, requiring extensive adjustments, checking, and monitoring behaviors. However, the degree of adherence to these other diets has never been rigorously assessed or compared. This article presents an investigation of dietary adherence and its predictors across five different restrictive dietary patterns.

### 1.1. Personality Characteristics Linked to Dietary Adherence

Observational studies have linked a variety of individual differences to adherence among weight-loss dieters specifically, including self-control and lower levels of emotional eating [6,7,8]. In addition, conscientiousness, openness to experience, and emotional stability (low neuroticism) have each been associated with better adherence in weight-loss intervention programs [9,10]. One of the few studies of adherence in a non-weight-loss sample explored personality traits among people with coeliac disease [11]. Here too, conscientiousness was positively associated with adherence.

### 1.2. Mental Health and Its Link to Dietary Adherence

Evidence suggests that mental health is associated with better adherence among weight-loss dieters [12,13]. This is also corroborated by studies of people on gluten-free diets, where depression, anxiety, or disordered eating is associated with poorer adherence to a gluten-free diet among people with coeliac disease [14]. The relationship between depression and poor adherence in coeliac disease has been confirmed in a recent meta-analysis [15], however, these authors note that the direction of this relationship remains unclear, as poor adherence would be expected to negatively affect health (at a minimum, physical health) in this population. The possibility that poor mental health is an outcome (rather than a cause) of poor adherence is corroborated by studies of supervised, formal, lifestyle-modification programs. People who successfully lose weight in such programs also report better wellbeing and fewer symptoms of disordered eating [16,17].

Another possibility is that poor mental health is attributable to the factors that led people to be on a weight-loss or gluten-free diet in the first place, e.g., obesity or chronic disease. There is good evidence, for instance, that much of the mental health burden of overweight and obesity is attributable to stigma [18,19]. People on other dietary patterns may also experience stigma and misunderstanding, for instance, people on a gluten-free diet may be socially excluded at restaurants or other social gatherings [20]. Here is where our approach can offer novel insight: By directly comparing adherence across rather than within dietary groups, we are able to identify predictors that are not specific to any one population and are thus more likely to be related to adherence itself.

### 1.3. The Neglected Role of Motivational Factors in Adherence

Prior research has rarely compared different dietary groups, and as a result, people’s motivation for their dietary choices is often assumed rather than directly investigated. For example, it is often taken as a starting point that a person with coeliac disease undertakes a gluten-free diet for health reasons, a person with obesity undertakes a weight-loss diet to lose weight, and so on. However, motivations for dietary behavior are diverse and, as with other health behaviors, likely to play a central role in adherence.

Motivational factors related to adherence have been predominantly explored in vegan and vegetarian samples. In particular, qualitative research has suggested that the strongly held ethical and moral beliefs among these groups is key to their long-term adherence [21,22,23]. Vegetarianism has also been found to be associated with progressive moral values more generally: integrity, empathy, being liberally minded, and self-sacrifice for the greater good [22]. One study found that, among vegetarians and vegans, people with an ethical motivation were more adherent than those with a health motivation [24]. This suggests that dietary motivations warrant further investigation across dietary groups as determinants of adherence.

Another motivational factor that has received a small amount of research attention specifically among vegetarians and vegans is social identity: When a dietary pattern is an enactment of a valued social identity, this may make adherence more likely [25]. Dietary patterns may become central to one’s self definition, particularly when surrounded by social networks or advocacy groups who follow the same dietary pattern [20,21,26]. One study found that social identification with one’s dietary pattern was particularly high among vegetarians and vegans [27], and another found preliminary evidence that this positively predicted adherence in this community [28]. Finally, the motivational factor that has received the most research attention to date is self-efficacy, or confidence in adhering to one’s dietary choices. Self-efficacy has been found to be important for weight loss and maintenance [29,30].

### 1.4. The Current Study

This study sought to provide the first comprehensive profile of dietary adherence and its predictors in a diverse sample following five different restrictive dietary patterns: vegan, vegetarian, paleo, gluten-free, and weight-loss. Our methodological approach included both (1) a qualitative component based on participants’ subjective understandings of the facilitators of and barriers to adherence, and (2) a quantitative component examining the psychosocial predictors of dietary adherence. In particular, we focused on three classes of variables that have previously been implicated in adherence research (in most cases, for one dietary group only): personality, mental health, and motivational factors.

This exploratory study had two goals:

1. To compare the degree of adherence between dietary groups in order to establish whether people following some restrictive dietary patterns are more adherent than others.
2. To investigate predictors of adherence across dietary groups to establish whether there are some psychosocial factors that may support adherence universally, regardless of which dietary pattern a person seeks to follow. We tentatively predicted that the importance of motivational factors may have been underestimated in prior work focusing on one dietary group at a time, relative to personality and mental health factors.

## 2. Materials and Methods

### 2.1. Participants and Design

Participants were 292 people following restrictive dietary patterns and were predominantly female (85.5%), Caucasian (84.6%), and of a healthy weight (59.4%). Participants’ ages ranged from 17 to 74 years (*M* = 31.44, *SD* = 12.99). Participants were recruited via advertisement on web forums, social media groups, and special interest groups for specific dietary groups. Snowball sampling and a university recruitment pool were also used. Recruitment continued until a minimum of 35 people per restrictive dietary group was reached. University students received course credit for participating; community members did not receive an incentive. All participants provided informed consent and the study was approved by the Human Research Ethics Committee at the University of Queensland (#16-PSYCH-MAP-02-JH).

To assess restrictive dietary pattern group membership, participants were first asked a free response question “Please describe your dietary pattern, in terms of what foods you choose to eat and which foods you choose to avoid (not eat at all) or minimize (eat less often).” Participant then self-categorized into a dietary group based on the question “Which of the following groups best describes your dietary pattern?” with response options “vegetarian”, “vegan”, “paleo”, “gluten free”, “on a weight loss diet”, “on an unrestricted diet” and “on another specific diet”. Participants who selected the final option were asked to provide the name of their specific diet. The free response questions were used to reclassify a small number of participants, e.g., a participant self-categorized as being on “another specific diet” but who described it as “a 2/5 diet for weight loss” was reclassified into the weight-loss diet group. Respondents who were on an unrestricted diet (*n* = 101) or on another type of restricted diet that had low frequency in this sample (*n* = 15, e.g., dairy and nut avoidance due to allergy) were excluded, to yield a final sample of 292.

Participants also reported length of time following their dietary pattern and were asked to specify any diet-related medical conditions. The majority of the sample had been following their dietary pattern for over 2 years (51.0%). A diet-related health condition was reported by a minority of participants (21.2%), but a majority of the gluten-free group (78.9%) reported a diet-related health condition, most commonly coeliac disease, followed by a gluten intolerance/allergy or suspected (but not biopsy confirmed) coeliac disease.

### 2.2. Measures of Adherence

#### 2.2.1. Adherence

Assessment of dietary adherence is difficult, and the gold standard is one-on-one evaluation with a trained dietitian [31]. However, given that this was not feasible in the survey format, we utilized two self-report measures of adherence to one’s dietary pattern: subjective adherence and measured adherence.

The subjective adherence measure was adapted from the Global Evaluation of Eating Behavior [32]. It included six items such as “I consistently ate my chosen dietary pattern during the past two weeks” measured on a scale from 1 “strongly disagree” to 7 “strongly agree”, α = 0.85. For comparability to the measured adherence measure, items were summed and then standardized to yield an overall subjective adherence scale with *M* = 0 and *SD* = 1 (range of −3.35–0.77).

Measured adherence was assessed using an adapted Food Frequency Questionnaire [33,34], which was revised and piloted with guidance from a dietitian (third author). Participants were asked “On average, in the previous month, how often did you consume the following food items, irrespective of portion size?” All participants were asked about 14 foods relevant to assessing adherence to multiple dietary patterns, drawn from current dietary guidelines [35]. For example, “Fish and seafood”, on a scale from 1 “Never” to 5 “Daily”. In addition, participants were asked about 5-10 items that assessed common forms of nonadherence in their specific dietary group (e.g., beer for the gluten-free group). For each of the five restrictive dietary patterns, measured adherence was calculated by summing responses to the subset of items that constituted nonadherence to their dietary pattern. Scores were reversed, scaled, and standardized to allow for direct comparison between groups, such that a person from the vegan group who indicated “Never” on all items containing animal products would receive the same (maximum) adherence score as a person from the gluten-free group who indicated “Never” on all items containing gluten. This yielded an overall measured adherence scale with *M* = 0 and *SD* = 1 (range of −4.34–0.99).

Subjective and measured adherence were moderately correlated (*r* = 0.50, *p* < 0.001). Given this, each analysis predicting adherence was conducted separately for both subjective and measured adherence.

Participants were also asked two open-ended questions about the barriers and facilitators of dietary adherence. These were: “Please describe what helps you to maintain your dietary pattern” and “Please describe any times when you struggle to maintain your dietary pattern”.

### 2.3. Measure of Personality Characteristics

#### 2.3.1. Self-Control

The 13-item Brief Self-Control Scale [36] was included. Participants rated items such as “People would say I have very strong self-discipline” on a scale from 1 “Not at all” to 5 “Very much”, *α =* 0.85.

#### 2.3.2. Emotional Eating

The Emotional Eating subscale from the Dutch Eating Behavior Questionnaire [37] is a 13-item measure on which participants rated items including “Do you have the desire to eat when you are emotionally upset” on a scale from 0 “Never” to 5 “Very often”, *α* = 0.93.

#### 2.3.3. Big Five

The 20-item Mini-IPIP [38] is a short version of the Five-Factor Model questionnaire [39], and was used to assess five personality factors on a scale of 1 “Very Inaccurate” to 5 “Very Accurate”: extraversion (“Am the life of the party”), agreeableness (“Feel others’ emotions”), conscientiousness (“Like order”), neuroticism (“Get upset easily”), and openness to experience (“Have a vivid imagination”). The scale is moderately reliable (*α* = 0.65–0.82 [38]), which was reflected in this study (*α* = 0.63–0.82).

### 2.4. Measures of Mental Health

#### 2.4.1. Disordered Eating Behaviors

The five-item screener version of the Eating Disorder Inventory [40] was included. This scale included items such as “I have the thought of trying to vomit in order to lose weight” rated on a scale from 0 “Never” to 4 “Always”. Three items screen for anorexia nervosa (*α*_AN_ = 0.53) and two items screen for bulimia nervosa (*α*_BN_ = 0.51). The mean of the five items was calculated to create a single indicator of the severity of disordered eating with better reliability than the subscales (*α* = 0.68).

#### 2.4.2. Depression, Anxiety, and Stress Symptoms

The Depression, Anxiety, and Stress Scale (DASS-21; [41]) was included. Participants rated the frequency of symptoms on the three subscales of depression (e.g., “I felt down-hearted and blue”), anxiety (e.g., “I felt scared without any good reason”), and stress (“I found it hard to wind down”). The scale has been found to be valid and reliable (*α* = 0.82–0.97; [42]), which was further supported by this study (*α* = 0.82–0.91).

### 2.5. Measures of Motivational Factors

#### 2.5.1. Dietary motivation

The Food Choice Questionnaire [43] was used to assess participants’ motivation to eat specific foods. Participants responded to 36 items on a scale from 1 “Not important at all” to 7 “Extremely important”. Mean scores were calculated for nine subscales: health (e.g., “Is nutritious”), mood (e.g., “Makes me feel good”), weight control (e.g., “Is low in calories”), natural content (e.g., “Contain no additives”), convenience (e.g., “Takes no time”), sensory appeal (e.g., “Tastes good”), price (e.g., “Is cheap”), familiarity (e.g., “Is like the food I ate when I was a child”), and ethical concern (e.g., “Is approved politically”). This study found good reliability for all of the subscales (*α* = 0.77–0.93) except the ethical concern factor (*α* = 0.63).

#### 2.5.2. Moral Foundations

In order to measure participants’ moral values, the 20-item moral foundation questionnaire was used [44,45]. This scale measured participants’ ratings of the importance of five specific moral domains: care (“Whether or not someone was cruel”), purity (“Whether or not someone did something disgusting”), loyalty (“Whether or not someone showed a lack of loyalty”), fairness (“Whether or not someone acted unfairly”), and authority (“Whether or not someone showed a lack of respect for authority”). However, reliability estimates were unacceptably low for the loyalty (*α* = 0.51) and fairness (*α* = 0.57) subscales. Therefore, in accordance with recommendations [46], the moral foundations questionnaire was used to calculate a single scale representing progressive moral values (the mean of the care and fairness subscales, *α* = 0.78, minus the mean of the loyalty, authority, and purity subscales, *α* = 0.83).

#### 2.5.3. Social Identification with Dietary Group

The In-group Identification Scale [47] is a 14-item measure that was used to assess social identification with one’s dietary group. Participants’ dietary pattern was piped into the questionnaire from their previous responses, such that the example items included the specific social category, e.g., “I feel a bond with others who are [vegan]” on a scale from 1 “strongly disagree” to 7 “strongly agree”, *α* = 0.93.

#### 2.5.4. Self-Efficacy

A validated single-item scale [48] was used to assess self-efficacy. Participants responded to the question “How confident are you that you will be able to stick to your dietary pattern for the next 90 days, or three months?” with response options ranging from 1 “Not at all confident” to 10 “Very confident”. This widely used short-form measure has been found to have comparable psychometric properties to longer self-efficacy scales.

#### 2.5.5. Demographics

Participants provided their age, gender, ethnicity, height, and weight (to calculate body mass index [BMI]).

### 2.6. Analysis Plan

#### 2.6.1. Qualitative Analyses

The open-ended questions regarding the barriers and facilitators of dietary adherence were subjected to two types of qualitative analysis by two independent coders. The first was an experienced qualitative researcher (fourth author) who used an inductive thematic analysis approach to identify dominant themes in the way that each group of participants justified adherence (or the lack of it) to their dietary patterns [49]. The analysis was grounded in the data and based on multiple examples (a bottom-up approach [50], rather than guided through a preconceived theoretical lens [49,51].

The second coder, who was blind to dietary groups and the hypotheses, used a frequency analytic approach [52]. First, themes in what participants described as facilitators or barriers to adherence (or both) were identified. Subsequently, the number of instances of each theme for each group was calculated so that this could be subjected to frequency analyses through chi-square tests, as a means of “confirming” the themes generated by the first coder, as well as to explore the frequency of the themes across all groups. There was a large overlap between the two qualitative approaches.

#### 2.6.2. Quantitative Analyses

Three stages of quantitative analyses were conducted. First, given that there are demographic differences between dietary groups [53], in stage one we first examined whether any demographic characteristics predicted adherence in an analysis of variance (ANOVA), which included gender, ethnicity, health condition, and dietary group as categorical predictors, and age and BMI as continuous predictors. In stage two, regression analyses were conducted to investigate which of the psychosocial variables from each category significantly predicted adherence in the overall sample. All of the potential predictors from each domain (personality, mental health, and motivational measures) were included simultaneously in a series of regression analyses predicting adherence. In stage three, a hierarchical regression model was run including the significant predictors from each domain-specific analysis. Variables that were no longer significant in the combined analysis were excluded from the final model.

## 3. Results

### 3.1. Qualitative Analyses

According to the frequency analysis, in the overall sample, the most common facilitators of adherence were ethical/moral concerns (51.6%), health (39.4%), and conscientiousness (15.9%). The most common barriers to adherence were inconvenience (37%) and a lack of willpower (31%). Examples of each theme, the frequency of each theme, and statistical comparisons between frequencies for each dietary group are provided in Table 1.

#### 3.1.1. Vegans: Diet Adherence as a Socio-Political Struggle

This group described their diet not as simply a dietary pattern, but as an ethical way of life:

*“Realizing the fact that meat comes from animals who suffer in order to feed people. Same applies to dairy. The entire meat industry is evil and I have no intention to support it.”*

(P142)

*“The ongoing desire to do no harm.”*

(P57)

It was common for vegans to construct diet adherence against a context of broader social and political struggles related to animal rights and environmental protection. Vegans reported few difficulties in adhering. Vegans were the group most likely to report ethical/moral concerns (80.5%) and identity (9.8%) as facilitators of adherence. Vegans did not frame their diet in terms of individual concerns, being the least likely to mention the facilitators of conscientiousness (8.1%) or weight loss (1.6%). Vegans were also least likely to report that a lack of willpower was a barrier to adherence (20.0%) and most likely to report that they experienced no barriers at all (25.2%).

#### 3.1.2. Vegetarians: Diet Adherence as Standing up for one’s Moral and Ethical Values

Similar to vegans, vegetarians commonly accounted for their diet adherence as a manifestation of their social ethics, rather than an individual lifestyle choice:

*“Reminding myself of the ethical reasons of why I am vegetarian definitely keeps me on track…Knowing that I am standing up for something that I believe in makes me feel proud.”*

(P26)

*“My belief system is about viewing animals as companions/to be respected in their own right not consumed as food. It is an important part of who I am.”*

(P36)

*“My environmental values. If I eat meat, it isn’t the same as it used to be. I feel very guilty and disgusted, and think about the animal alive and yada yada yada. So I avoid meat for the discomfort I experience now.”*

(P9)

In certain instances, nonadherence was linked to guilt and disgust (P9). Identifying strongly with certain social groups and their values (e.g., vegetarianism) meant that people anticipated unpleasant physiological and psychological responses if they were to breach the groups’ code of moral conduct (i.e., nonadherence). These patterns were mirrored in the frequency analysis, where the vegetarian group were the second most likely to mention ethics/morality (46.7%) and identity (8.9%) as facilitators. They also had the second highest frequency of people reporting no barriers to adherence (15.6%). Vegetarians were least likely to report health as a facilitator of adherence (11.1%).

#### 3.1.3. Paleo: Diet Adherence as Personal Preference and Benefit

In contrast to vegetarians and vegans, people following a paleo dietary pattern accounted for adherence in more individualistic terms. For example, paleo participants mainly reported that adherence was related to personal satisfaction, health benefits, and subjective preference:

*“Getting into a routine and feeling benefits i.e., more energy, weight loss, clearer skin etc.”*

(P184)

*“I don’t need help. I no longer crave processed, scary foods.”*

(P187)

*“I feel (and look) so much better when I stick to paleo eating that it’s my strongest motivation.”*

(P185)

Participants reported both physical (look better, weight loss) and psychological benefits (feel better) as adherence motivators. Ideological elements are also evident in participants’ accounts, albeit different to vegan and vegetarian accounts. For example, P187 refers to nonpaleo foods as “processed” and “scary.” The paleo group were most likely to mention enjoyment as a facilitator (17.9%), and second most likely to mention health as a facilitator (53.8%). On the other hand, they were also the second most likely to report lack of willpower as a barrier (41.0%), suggesting that this individualistic conceptualization of one’s dietary choices might also expose oneself to individual weakness.

#### 3.1.4. Gluten Free: Diet Adherence as the only Viable Option

Gluten-free dieters’ accounts focused on avoiding the adverse effects experienced when consuming products that contained gluten:

*“I get violently ill when I eat gluten.”*

(P217)

*“Gluten causes pain. I don’t like pain. I don’t eat gluten.”*

(P218)

*“I vomit 3 h after ingesting gluten, so it’s not a hard decision to avoid it.”*

(P233)

Although other groups also spoke about health as a motivator, the gluten-free group was distinct in its focus not on the benefits of adherence, but rather on the negative consequences of nonadherence such as pain, unpleasant symptoms, and physical harm. Moreover, while people in other dietary groups describe themselves as agents of their diet choices, gluten-free people positioned themselves as lacking choice. In line with this, people on the gluten-free dietary pattern were most likely to mention health (68.8%) as a facilitator. Interestingly, they were also most likely to mention inconvenience (48.6%) as a barrier to adherence.

#### 3.1.5. Weight-Loss Diet: Adherence as Personal Goal of Self-Discipline

Adherence among people on a weight-loss diet was primarily defined through individualistic repertoires:

*“Regularly maintenance and reassessing diet and dietary needs. Mindful action daily.”*

(P273)

*“looking at myself in the mirror and also eating foods outside of my diet plan makes me feel horrible and guilty so that helps as well.”*

(P254)

*“The fact that I’ve started and that I should stick to it.”*

(P247)

Participants on a weight-loss diet discussed adherence in terms of observed or expected benefits (e.g., happiness, body image). For most people in the weight-loss group, however, adherence was not linked to group-based values (e.g., ethics) or even health issues (e.g., energy or pain), but was presented as an obligation, a process of constant self-reminders or an act of self-discipline. Weight-loss dieters were, unsurprisingly, the most likely to mention weight loss as a motivation (22.9%). They were also the most likely to mention lack of willpower (68.6%) and mood/emotion (22.9%) as barriers to adherence.

### 3.2. Quantitative Analyses

In two ANOVA tests that included demographic characteristics, only dietary group significantly predicted either measured or subjective adherence. Therefore, demographic variables were not included as covariates in any of the analyses that follow.

**Figure 1:**
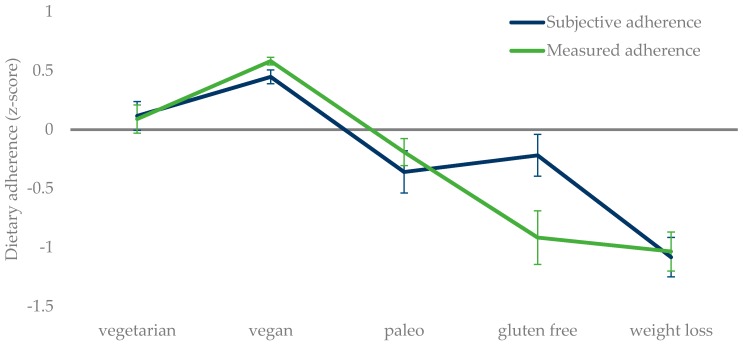
Adherence to one’s dietary pattern differed between dietary groups. Note. Error bars represent standard errors.

The dietary groups differed significantly in their average subjective adherence, *F*(4,275) = 25.02, *p* < 0.001, and in their average measured adherence, *F*(4,284) = 47.03, *p* < 0.001. As seen in Figure 1, the vegan dietary group had the highest average adherence on both measures, while the weight-loss group had the lowest average adherence on both measures. In terms of the divergence between the two measures of adherence, a series of planned simple comparisons indicated that these were significantly different for two out of the five dietary groups: the vegan group scored significantly higher on measured adherence than subjective adherence, *t*(123) = 2.55, *p* = 0.012, whereas the gluten-free group scored significantly lower on measured adherence than subjective adherence, *t*(34) = −2.70, *p* = 0.011.

#### 3.2.1. Personality Characteristics

Big five, self-control, and emotional eating were simultaneously added as predictors in regression models predicting adherence. Only one emerged as a significant predictor of measured adherence: emotional eating, β = −0.18, *p* = 0.018, *F*(7,238) = 1.74, *p* = 0.102, *R*^2^ = 0.05. Similarly for subjective adherence, only emotional eating was a significant predictor, β = −0.16, *p* = 0.035, *F*(7,238) = 2.22, *p* = 0.033, *R*^2^ = 0.06. In both cases, emotional eating negatively predicted adherence.

#### 3.2.2. Mental Health

Among disordered eating, depression, anxiety, and stress, only disordered eating was a significant predictor of measured adherence, β = −0.25, *p* = 0.001, *F*(4,262) = 3.27, *p* = 0.012, *R*^2^ = 0.05. Similarly, for subjective adherence, only disordered eating (β = −0.21, *p* = 0.001) was a significant predictor, *F*(4,262) = 5.34, *p* < 0.001, *R*^2^ =0.08. In both cases, disordered eating was associated with poorer adherence.

#### 3.2.3. Motivation

The nine subscales of the Food Choice Questionnaire, moral foundations, social identification and self-efficacy were predictors in the regression. Measured adherence was predicted by health motivation (β = 0.15, *p* = 0.025), natural content motivation (β = −0.13, *p* = 0.046), weight control motivation (β = −0.15, *p* = 0.021), self-efficacy (β = 0.16, *p* = 0.006), and social identification with one’s dietary group (β = 0.43, *p* < 0.001), *F*(12,235) = 10.54, *p* < 0.001, *R*^2^ = 0.35. Subjective adherence was predicted by mood motivation, β = −0.14, *p* = 0.027, self-efficacy, β = 0.38, *p* < 0.001 and social identification with one’s dietary group (β = 0.29, *p* < 0.001), *F*(12,235) = 11.40, *p* < 0.001 *R*^2^ = 0.37. These effects were such that people whose eating choices were motivated by their mood, natural content, or the desire for weight control had significantly poorer adherence. Those with greater self-efficacy, those motivated by health, and those who strongly identified with their dietary group had significantly better adherence.

#### 3.2.4. Final Predictive Model of Dietary Adherence

The final models are presented in Table 2. Of the five factors that predicted measured adherence in the domain-specific regression analyses above, only three were significant and retained in the final combined model: weight control motivation (β = −0.18, *p* = 0.001), self-efficacy (β = 0.16, *p* = 0.004), and social identification (β = 0.41, *p* < 0.001). Together these variables explained 30% of the variance in measured adherence.

**Figure 2:**
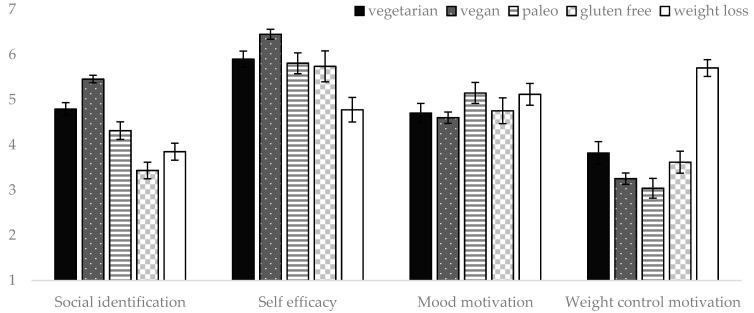
Differences in the average level of endorsement of each correlate of measured and/or subjective adherence between dietary groups. Note. Error bars represent standard errors. Self-efficacy has been converted to a seven-point scale for graphing purposes, but was a ten-point scale in the analyses.

Of the seven factors that predicted subjective adherence in the domain-specific regression analyses above, only three were significant and retained in the final combined model: mood motivation (β = −0.11, *p* = 0.027), self-efficacy (β = 0.41, *p* < 0.001), and social identification (β = 0.33, *p* < 0.001). Together, these variables explained 35% of the variance in subjective adherence. The significant predictors of adherence are presented for each dietary group in Figure 2.

## 4. Discussion

### 4.1. Summary of the Findings

The qualitative analyses revealed stark differences between dietary groups in perceived facilitators of and barriers to adherence. For vegetarians and vegans, adherence was tied to broader sociopolitical, anti-systemic struggles, and presented as a core part of one’s social identity. In the other three groups, these ideological patterns were not apparent. Participants following a gluten-free diet constructed adherence in terms of necessity and symptom avoidance. In contrast, participants in the paleo and weight-loss dietary groups drew from more individualistic repertoires of personal motivation and wellbeing; for them, maintaining their diet was a personal concern that was not linked to any specific social identity, and attempts to adhere to their dietary patterns were grounded in personal strength and restraint.

The quantitative data indicated that the dietary groups also differed in their degree of adherence, with both subjective and measured adherence being highest in the vegan group and lowest in the weight-loss group. Only four predictors, all motivational, explained a significant proportion of the variance in subjective or measured adherence in the final model. In order of variance explained, these were social identification with one’s diet group, self-efficacy, weight control motivation, and mood motivation.

### 4.2. Implications

The role of social identity in supporting good adherence emerged from both the qualitative and quantitative analyses. Specifically, vegan and vegetarian groups were the only participants to nominate identity as a facilitator of adherence in their free-responses. Subsequently, they also reported the strongest social identification with their dietary group in the quantitative measures, and this emerged as a strong predictor of both subjective and measured adherence. Indeed, a follow-up analysis found that dietary group was no longer significantly related to either subjective or measured adherence once social identification was added to the model. This was not true for any of the other predictors of adherence, which speaks to the central role of social identification in accounting for these differences between dietary groups. When a dietary pattern becomes a positive and meaningful part of one’s identity, adherence is no longer a chore that requires willpower and restraint, instead, it becomes an enactment of one’s values; an expression of the self [25,54]. In the words of one vegan participant: “eating non-vegan food would be betraying myself.” By contrast, people following a weight-loss or gluten-free diet tended not to incorporate their dietary pattern as a meaningful or positive part of their identity. Indeed, previous studies have suggested that identifying as overweight is actively harmful to mental health, in part because of the stigmatized and negative nature of this group membership [18]. One implication of this finding is that supporting people to find positive ways to self-define in terms of their dietary pattern may be a promising step forward for interventions, including to support people striving for healthy weight loss.

One consistent finding across the qualitative and quantitative analyses was the central role of mood motivation, or using food as a means to regulate one’s mood. This was a risk factor for poor adherence, and was particularly prominent among people following a weight-loss or paleo dietary pattern. Interestingly, it was not emotional dysregulation in general that was a risk factor, as neither the personality trait of neuroticism nor symptoms of psychological distress were significant predictors of adherence. This suggests the need for interventions to attend to the specific role of eating behavior as a mood control strategy (even among people in good mental health more generally) in order to improve dietary adherence.

Interestingly, the utility of being motivated by weight loss seemed to differ between the qualitative and quantitative analyses. People on a weight-loss diet reported that this motivation was a facilitator of adherence, however, in the quantitative analyses weight-loss motivation predicted poorer adherence (both subjective and measured). This accords with previous research, which has found that being motivated by weight loss, rather than health, may put people at risk of weight gain and disordered eating [55]. It appears, however, that the downsides of weight-loss motivation may not be apparent to dieters and thus could be a useful target for intervention. Strengthening other, more positive motivators such as health, values, or identity may result in better outcomes for weight-loss dieters.

Self-efficacy was a strong predictor of adherence in the quantitative analyses, and was highest in the vegan group. Consistent with this, in the qualitative analyses, the vegan group were least likely to report that a lack of willpower was a barrier to adherence and most likely to explicitly state that they experienced no barriers at all to adherence. The vegan group were distinct in their use of evocative language to emphasize their confidence in adhering to their dietary choices: “eating a steak would be as absurd as eating cardboard”; “I never have struggled with [being vegan] and I never will”. The importance of self-efficacy suggests that interventions that build confidence in one’s capacity for dietary adherence may be indicated (for instance, Motivational Interviewing [56,57]).

Noteworthy too are the factors that were not found to predict adherence in the present study. For example, depression, disordered eating, and self-control have been linked to adherence previously [6,58,59] but were non-significant here. There are several reasons why these may not have emerged in the current analysis. First, previous studies have focused predominantly on weight-loss dieters, a group for whom adherence is likely to rest more strongly on such individual traits, given their much lower endorsement of social identity and values-based dietary motives. In addition, previous reviews have indicated that the direction of the relationship between these factors and adherence is not yet clear [15]. Our data are consistent with prior arguments that mental ill-health, for instance, may be secondary to stigma associated with obesity rather than a cause of poor dietary adherence.

One surprising finding of this study was the relatively poor adherence among people on a gluten-free diet. This is despite the fact that this group described adherence as a medical necessity and positioned themselves as lacking choice or agency in their dietary pattern. Previous studies have typically found somewhat higher adherence in participants with biopsy-confirmed coeliac disease (although studies have varied widely [60]). Approximately half of this sample had a coeliac diagnosis, and the majority of the remainder reported following a gluten-free diet for health reasons (most commonly, suspected but unconfirmed coeliac disease, gluten intolerance, or allergy). Previous studies on adherence in this population have also tended to utilize the Coeliac Dietary Adherence Test [61], which is a self-report measure that focuses on the gastrointestinal symptoms secondary to nonadherence in coeliac disease. To investigate whether these differences might account for our results, post hoc analyses were conducted to assess whether a coeliac disease diagnosis was related to higher gluten-free diet adherence. Interestingly, coeliac disease was associated with higher subjective adherence (*r* = 0.41, *p* = 0.015), but not measured adherence (*r* = 0.24, *p* = 0.150). This is consistent with the general pattern in these data whereby the gluten-free diet group tended to over-estimate their adherence, relative to other groups. One implication of this research, then, is that although people with coeliac disease tend to accept that a gluten-free diet is a medical necessity for them, this does not necessarily translate into good (measured) adherence. Interventions to increase self-efficacy or positive diet-related identity may warrant further investigation for this population.

### 4.3. Limitations

As with any study, there are limitations to note of this project. First, these data are cross-sectional, and so caution is warranted in inferring the direction of these relationships. The sample is also predominantly young, relatively healthy women and so it is unclear yet whether the findings generalize to older adults whose dietary choices may be related to chronic health conditions. Finally, although this study included multiple measures of adherence, it is nevertheless difficult to obtain valid indicators of long-term food intake, and so like many studies of diet and nutrition, these findings are qualified by the limitations of these self-report measures.

## 5. Conclusions

This study investigated dietary adherence in people following five different kinds of restrictive dietary patterns. Although adherence is notoriously low for people on weight-loss diets, we found that this was not generalizable to people on other dietary patterns, with vegans and vegetarians showing high subjective and measured adherence. Drawing upon both qualitative and quantitative evidence, this study found that confidence in and commitment to one’s dietary pattern (i.e., self-efficacy and social identification) were the strongest predictors of adherence. By contrast, being motivated by mood or weight control tended to be associated with poorer adherence. Overall, these findings point to the central role of motivational factors and suggest that strategies to support dietary self-efficacy and positive diet-based identities may have promise in facilitating dietary adherence.

## Figures and Tables

**Table 1:** Frequency of each theme in qualitative description of barriers and facilitators of adherence among each dietary group.

Theme *Example*	Vegetarian (*n* = 48)	Vegan (*n* = 128)	Paleo (*n* = 42)	Gluten Free (*n* = 38)	Weight Loss (*n* = 36)	Pearson χ (*df*)	*p* Value
**Facilitators**							
Ethical/moral	46.7% ^a^	80.5% ^b^	12.8% ^c^	8.6% ^c^	42.9% ^a^	92.04 (4)	<0.001
*“It’s SO easy to be vegan once you have made the ethical connection. Once you see animals as living being creatures who feel pain and joy. Animal products and by-products don’t look like food to me. Eating a steak would be as absurd as eating cardboard.”*							
Health	11.1% ^a^	34.1% ^b^	53.8% ^c^	68.6% ^c^	48.6% ^b, c^	33.64 (4)	<0.001
*“I feel very unwell if I do not maintain my dietary pattern so a feeling of wellness after eating is what helps me to maintain my diet.”*							
Weight loss	2.2% ^a, b^	1.6% ^b^	12.8% ^a, c^	2.9% ^a, b^	22.9% ^c^	26.21 (4)	<0.001
*“The thought of losing fat fast and being lean”*							
Identity	8.9% ^a, b^	9.8% ^a^	0.0% ^b^	0.0% ^a, b^	0.0% ^a, b^	11.06 (4)	0.026
*“I identify as Vegan above all other things so eating non-vegan food would be betraying myself.”*							
Enjoyment	17.8% ^a^	8.1% ^a, b^	17.9% ^a^	2.9% ^b^	2.9% ^b^	10.42 (4)	0.034
*“Coming to the realization that healthy foods are actually delicious and more satisfying than junk food and sweets.”*							
Conscientiousness	24.4% ^a^	8.1% ^b^	20.5% ^a^	20.0% ^a^	22.9% ^a^	10.35 (4)	0.035
*“Being prepared, reading absolutely every label (this happens in the shopping process so I don’t have Gluten products in the cupboard). Check everything I put into my mouth.”*							
**Barriers**							
Lack of willpower	26.7% ^a, b^	20.3% ^a^	41.0%^b^	25.7% ^a, b^	68.6% ^c^	32.31 (4)	<0.001
*“When I have cravings it can take mental strength.”*							
Mood/emotion	0.0% ^a^	8.1% ^b, c^	17.9% ^c, d^	2.9%^a, b^	22.9% ^d^	17.47 (4)	0.002
*“Whenever I am feeling down/depressed as a result of other life stressors I have generally used food as a coping mechanism so it is hard to stay strict during these times”*							
Inconvenience	44.4% ^a^	39.8% ^a^	33.3% ^a^	48.6% ^a^	11.4% ^b^	13.52 (4)	0.009
*“Sometimes it is difficult when traveling and I have at certain times not followed my dietary patterns as a necessity.”*							
None	15.6% ^a, b^	25.2% ^a^	12.8% ^a, b^	14.3% ^a, b^	2.9% ^b^	11.11 (4)	0.025
*I never have struggled with it and I never will.*							

Note. The frequency of each theme in each dietary group was compared using a Chi-square analysis. For each theme, dietary groups with different subscripts had significantly different frequencies of that theme at *p* < 0.05.

**Table 2:** Final regression models predicting dietary adherence (one regression analysis for each dependent variable).

	β	*SE*	*p* Value	Semi-Partial *r*^2^	*F*(*df*)	Model *R*^2^
**Measured adherence**					38.67 (3,271)	0.30
*Predictors*						
Self-efficacy	0.16	0.03	0.004	0.02		
Social identification	0.41	0.04	<0.001	0.16		
Weight control motivation	−0.18	0.03	0.001	0.03		
						
**Subjective adherence**					48.62 (3,270)	0.35
*Predictors*						
Self-efficacy	0.41	0.02	<0.001	0.15		
Social identification	0.33	0.04	<0.001	0.10		
Mood motivation	−0.11	0.03	0.027	0.01		

Note. To reduce the risk of Type I error, variables were only retained in this model if they were significant predictors of adherence in both the domain specific analyses (the three domains of personality, mental health, and motivation were investigated) as well as in the combined analysis.
